# Exosomes Derived From Human Adipose-Derived Stem Cells Inhibit Lipogenesis Involving Hedgehog Signaling Pathway

**DOI:** 10.3389/fbioe.2021.734810

**Published:** 2021-08-31

**Authors:** Ziwan Ji, Zhongming Cai, Shuming Gu, Yucang He, Zikai Zhang, Tian Li, Qing Wei, Jingping Wang, Chen Ke, Liqun Li

**Affiliations:** ^1^ Department of Plastic Surgery, The First Affiliated Hospital of Wenzhou Medical University, Wenzhou, China; ^2^ The Affiliated Xiangshan Hospital of Wenzhou Medical University, Wenzhou, China

**Keywords:** adipose-derived stem cells, adipogenic differentiation, exosomes, hedgehog signal pathway, lipogenic

## Abstract

Since obesity impairs wound closure and adipose-derived exosomes (ADEs) regulate wound healing in clinical applications, we hypothesized that ADEs may inhibit adipogenesis of adipose-derived stem cells (ADSCs) to reduce the adverse effects of obesity on wound healing. Hedgehog (Hh) signaling has been previously shown to inhibit adipogenesis in ADSCs. The present study aimed to determine the role of ADEs in the adipogenesis of ADSCs and the Hh signaling pathway. ADSCs collected from human adipose tissues were co-cultured with ADEs and treated with an adipogenic inducer. qRT-PCR showed that ADEs could inhibit adipogenic differentiation of ADSCs and activate Hh signaling. The differences in the mRNA expression profiles of genes related to Hh signaling between the groups that were exposed to either high fat or low fat indicated that increased Hh signaling activation is necessary but not sufficient to inhibit adipogenic differentiation in the ADSC differentiation process. The Hh signaling pathway can be activated effectively by ADEs, especially during high-fat exposure after treatment with ADEs. Oil Red O staining of adipocytes suggested that ADEs inhibited not only adipogenic differentiation, but also lipogenesis in ADSCs. Overall, targeted activation of Hh signaling by ADEs reduced lipid accumulation in ADSCs and may be explored for clinical applications.

## Introduction

Obesity, characterized by an excess of adipose mass, is a major health problem of the 21st century (Haslam and James, 2005). It has been found to be associated with impaired wound healing in animal models (Egbert et al., 2020; Rock et al., 2019) and patient-cohort studies (Enser and Avery, 1984; Goodson and Hunt, 1986). Impaired wound closure because of obesity is a complex problem involving many factors. Microenvironment alterations caused by obesity have been reported as a potential contributing factor, as they may lead to a reduced plasticity of adipose-derived stem cells (ADSCs).

In 2001, ADSCs were successfully isolated from human fat tissues and were shown to have the potential for adipogenic, osteogenic, and chondrogenic differentiation (PA et al., 2001). ADSCs are an ideal cell type for clinical application owing to their ease of isolation and culture and abundant sources. Because of their self-replication and multidirectional differentiation properties, ADSCs have been successfully used in stem cell therapy strategies for tissue engineering and regenerative medicine. In addition, *in vitro* and *in vivo* experiments and clinical studies have demonstrated that ADSCs promote skin wound healing (Pu et al., 2017; Shin et al., 2017; Tao et al., 2016).

Exosomes are microvesicles 30–150 nm in diameter that carry proteins, lipids, and nucleic acids (Colombo et al., 2014). Growing evidence suggests that exosomes also carry signaling molecules and mediate intercellular long- and short-distance communication between different cell types in the body (McGough and Vincent, 2016; Luo et al., 2017). In addition, exosomes have been used as drug-delivery vehicles for diverse clinical applications (Kourembanas, 2015). For example, adipose-derived exosomes (ADEs) have been used to promote cutaneous wound healing (Ma et al., 2019; An et al., 2021).

Hedgehog (Hh) is a secreted morphogen involved in the development of mesenchymal stem cells. It was first discovered by Nusslein-Volhard et al. in 1980 by screening genes affecting *Drosophila* development (Nüsslein-Volhard and Wieschaus, 1980). Genetic studies have demonstrated that Hh effectively inhibits fat formation (Pospisilik et al., 2010). Hh is post-translationally modified, presenting a cholesterol molecule in the C-terminal region and a palmitate in the N-terminal region, which permit its anchoring to lipid membranes in the cell (Porter et al., 1996; Pepinsky et al., 1998). The Hh signaling pathway is initiated by the binding of Hh-secreted ligands to the extracellular domain receptor Patched (PTC), a conserved 12-pass transmembrane protein receptor. PTC inhibits the activity of smoothened (SMO), which is prevented by the interaction between Hh and PTC. This permits the activation of the transcription factor Gli, which then migrates to the nucleus, where it regulates several transcriptional processes (Goetz et al., 2009; Ruat et al., 2014). Moreover, the lipid resistance of Hh signaling has been clearly demonstrated in mouse cells *in vitro* (Cousin et al., 2007). A recent study has shown that Hh signaling had a relatively mild effect in mice fed a normal diet but significantly inhibited the accumulation of adipose tissue in mice fed a high-fat diet (Shi and Long, 2017). In addition, Hh also inhibits adipocyte differentiation (Shi and Long, 2017). Exosomes have been suggested to mediate the long-term effects of Hh signaling (Ng et al., 2014), and it has been reported that exosome-bound Hh can activate downstream signaling pathways and the transcription of target genes (Bischoff et al., 2013). Furthermore, exosomes derived from ADSCs have been shown to play a positive role in the treatment of trauma (Zhang et al., 2018) and inflammation (Cho et al., 2018; Patel et al., 2018). However, the exact mechanisms by which ADEs carrying Hh influence adipocyte differentiation and lipid production remain elusive.

We hypothesized primarily that ADEs inhibit adipogenesis in ADSCs to reduce the adverse effects of obesity on wound healing. The present study aimed to explore the role of ADEs in adipogenic differentiation and lipid accumulation in ADSCs and in the Hh signaling pathway.

## Materials and Methods

### Isolation of Human ADSCs

ADSCs were isolated from adipose tissue obtained from a young healthy woman undergoing liposuction of the abdomen. The oil components and the blood layer were discarded. An equal volume of collagenase I (Gibco, Madison, WI, United States) was added to the adipose tissue and gently blown. The mixture was placed in an oscillating chamber set to 280 rpm for 1 h at a constant temperature of 37°C. Equal volumes of Dulbecco’s modified Eagle medium (DMEM) were then added to the mixture to terminate the digestion. The mixture was filtered through a 100-mesh filter to remove oil and a 200-mesh filter to remove impurities. The filtrate was centrifuged at 1,200 rpm for 5 min, after which the supernatant was discarded, and the pellet was resuspended in DMEM and seeded into six-well plates.

### Adipose-Derived Exosome Isolation

Fetal bovine serum (FBS) was depleted of exosomes *via* ultracentrifugation at 100,000 × g for 70 min and subsequent straining through a sterile 0.22 μm-pore membrane filter. The cells were cultured in medium containing 10% exosome-free FBS for 24 h. Then, the cell culture medium was collected and centrifuged at 2,000 × g for 10 min to remove dead cells and twice at 10,000 × g for 15 min to remove cell debris and apoptotic bodies. The supernatant was passed through a 0.22 μm filter to remove debris and microvesicles and then ultracentrifuged at 100,000 × g for 70 min. The supernatant was discarded, and the sediment was resuspended in phosphate-buffered saline (PBS) and ultracentrifuged at 100,000 × g for 70 min to remove contaminating proteins. The pelleted vesicles were resuspended in 100 μL PBS. ADEs were defined as exosomes isolated from the conditioned medium of the ADSCs.

### Transmission Electron Microscopy

The pelleted ADEs were fixed with 2.5% glutaraldehyde in 0.1 M sodium cacodylate buffer (pH 7.4). The samples were post-fixed with cacodylate-buffered 2% OsO4. After rinsing in PBS (pH 7.4), the samples were successively dehydrated in a graded ethanol series and acetone. The samples were embedded in Epon 812 and cut into 50–70 nm super-thin slices. The slices were then stained with acetate and lead citrate, and the ADEs were examined *via* TEM using a JEM-1200EX microscope (JEOL, Tokyo, Japan).

### Nanoparticle Tracking Analysis

NTA was performed using a Zetaview instrument (Particle Metrix, Germany), according to the manufacturer’s protocol. The particle size distribution and concentration of all types of nanoparticles with diameters of 10–2000 nm could be analyzed rapidly and automatically *via* NTA.

### Western Blot Analysis

Cell lysates were prepared using radioimmunoprecipitation assay buffer (Solarbio, Beijing, China) supplemented with phenylmethanesulfonylfluoride fluoride (Solarbio).

Protein concentrations of exosomes or cell lysates were determined using the bicinchoninic acid assay, and equal amounts of proteins (20 μg) were separated on a 10% sodium dodecyl sulfate-polyacrylamide gel and electrotransferred onto a polyvinylidene difluoride membrane. The membranes were blocked in Tris-buffered saline with Tween 20 containing 5% fat-free dry milk and then incubated with the corresponding primary antibody overnight at 4°C followed by incubation with a horseradish peroxidase (HRP)-conjugated secondary antibody for 1 h. Proteins were detected using enhanced chemiluminescence reagents. The primary antibodies used are as follows: CD9 (Beyotime Biotechnology, Nantong, China), CD63 (Beyotime Biotechnology), Alix (Abcam, Cambridge, United Kingdom), and GM130 (Abcam, Cambridge, United Kingdom). The secondary antibody used was HRP-labeled Goat Anti-Rabbit IgG(H + L) (Beyotime Biotechnology).

### Treatment With ADEs

**FIGURE 1:**
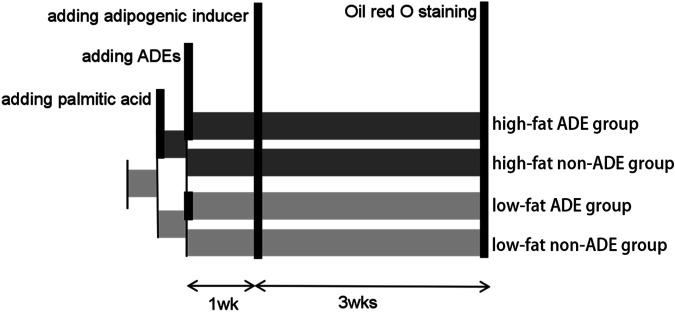
Schematic of the experimental design. ADSCs were cultured in an exosome-free medium. In the high-fat exposure group, 50 μM palmitic acid dissolved in bovine serum albumin was added to ADSCs to create a high-fat environment. ADSCs were co-cultured with ADEs in the ADE group. After 1 week, an adipogenic inducer was added to the media.

As shown in Figure 1, the ADSCs were divided into high-fat and low-fat exposure groups, both of which were treated with exosome-free medium. The high-fat exposure group was cultured in the presence of palmitic acid at a concentration of 50 μM to simulate exposure to high fat. Each of the two groups was then further divided into two subgroups according to the presence or absence of ADEs, resulting in a total of four groups, namely low-fat non-ADE, low-fat ADE, high-fat non-ADE, and high-fat ADE.

### Adipogenic Differentiation

After treating the ADSCs with or without ADEs for 1 week, the growth medium was replaced with human ADSC adipogenic differentiation basal medium (Cyagen, Santa Clara, CA, United States): 1) phase I induction (3 days)–adipogenic differentiation basal medium A (175 ml) with FBS (20 ml), insulin (400 µl), isobutylmethylxanthine (200 µl), dexamethasone (200 µl), penicillin-streptomycin (2 ml), L-glutamine (2 ml), and rosiglitazone (200 µl); and 2) phase II differentiation (1 day): adipogenic differentiation basal medium B (175 ml) with FBS (20 ml), insulin (400 µl), penicillin-streptomycin (2 ml), and L-Glutamine(2 ml). Phases I and II were carried out alternately for 3 weeks to induce adipogenic differentiation.

### qRT-PCR

Total RNA was isolated using an RNA Extraction Kit (Solarbio) and reverse-transcribed into cDNA using an iScript cDNA synthesis kit (Bio-Rad, CA, United States). Fast-start SYBR Green (Bio-Rad) was used for qPCR in a Step-One machine (Applied Biosystems, MA, United States). The nucleotide sequences of the primers used are listed in Table 1. Each RNA sample extracted from one well of the cell culture plate was considered a biological replicate.

**TABLE 1:** Nucleotide sequences of the primers.

Gene	Primer F/R	Sequence (5ʹ to 3ʹ)
*Shh*	F	GTC​TCC​TCG​CTG​CTG​GTA​TG
*Shh*	R	TTG​GGG​ATA​AAC​TGC​TTG​TAG​G
*Ptc*	F	GCC​TTG​GCT​GTG​GGA​TTA​AAG
*Ptc*	R	CTT​CTC​CTA​TCT​TCT​GAC​GGG​T
*Smo*	F	CGT​GCG​TGA​CCC​GTT​GTA​T
*Smo*	R	ATG​TCC​ATC​GTC​CAT​TCC​AG
*Gli2*	F	CAC​CTG​CAT​GCT​AGA​GGC​AAA
*Gli2*	R	AGA​AGT​CTC​CAT​CTC​AGA​GGC​TCA​TA
*Pparγ*	F	GGA​AAG​ACA​ACG​GAC​AAA​TCA​C
*Pparγ*	R	TAC​GGA​TCG​AAA​CTG​GCA​C
*C/EPBα*	F	GAA​TCT​CCT​AGT​CCT​GGC​TC
*C/EPBα*	R	GAT​GAG​AAC​AGC​AAC​GAG​TAC

### Oil Red O Staining

The cells were washed with PBS and fixed in 4% paraformaldehyde for 15 min. The cells were then stained with an Oil Red O working solution (3:2, Oil Red O saturated solution in isopropanol: distilled water) for 30 min at room temperature and washed three times with PBS. Staining was visualized using an inverted microscope (Olympus, Tokyo, Japan). For quantitative assessment, the stained cells were eluted with 100% isopropyl alcohol and quantified by spectrophotometric absorbance at 520 nm on an automatic microplate reader (Dynex Technologies, Gentilly, VA, United States) against a blank (100% isopropyl alcohol).

### Determination of Cell Size and Lipid Accumulation

To examine lipid accumulation, the cells were fixed with paraformaldehyde and stained with Oil Red O. Under the microscope, three sections of each well in five random fields were used for the semiquantitative analysis of the relative mean area and number of lipid droplets using ImageJ (available at http://imagej.nih.gov/ij/). The adipocytes were outlined, and the area was calculated using the Analyze tool, after which the mean ± SD was calculated. To evaluate cell morphology, more than 300 cells were randomly selected and morphologically inspected under the microscope. To determine the cell number, the cells present in three random images that had been previously acquired using the microscope were analyzed in each condition.

### Cell Proliferation Assay

ADSCs were plated in six-well plates at a density of 4,500 cells per cm^2^. At subconfluence (2 days later), adherent cells were dissociated with 0.25% trypsin-ethylenediaminetetraacetic acid and counted using a brightfield cell counter (DeNovix, Wilmington, DE, United States).

### Statistical Analysis

Statistical differences between groups were analyzed by Student’s *t*-test. Statistical significance thresholds are as follows: *p ≤ 0.05, **p ≤ 0.01, and ***p ≤ 0.001.

## Results

### Morphological and Multidirectional Differentiation of Human ADSCs

**FIGURE 2:**
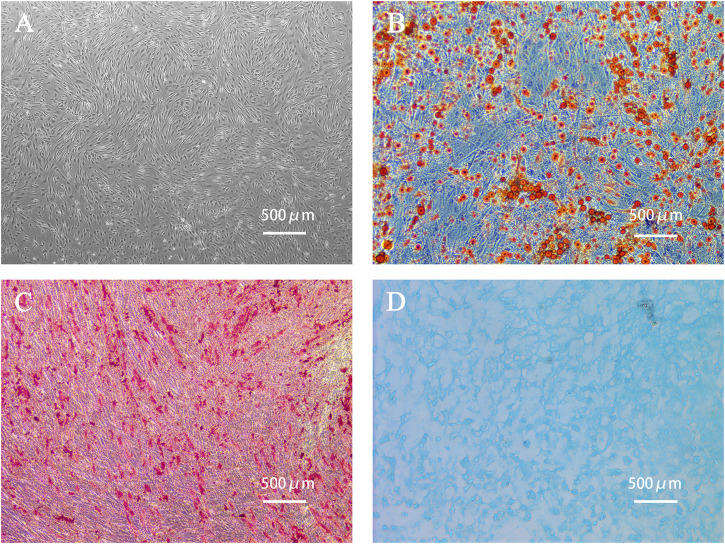
Human adipose-derived stem cells (ADSCs) and cell morphology after differentiation. **(A)** The fourth generation of ADSCs grew in colony vortex. **(B)** Human ADSCs were stained with Oil red O after adipogenic differentiation. The red parts are lipid droplets. **(C)** Alizarin red staining after osteogenesis induction and differentiation of human ADSCs. The red parts are calcium nodules. **(D)** Alcian blue staining after induction and differentiation of human ADSC chondroblast. The blue part is the endoacidic mucosaccharide in cartilage tissue.

The primary ADSCs obtained from the adipose tissue samples of a young healthy womanbegan to proliferate 4 h after seeding them and grew rapidly after adhering to the well surface. After 24 h of incubation, the morphology of the adherent cells changed from round to spindle-shaped. Cells of the fourth generation grew well in a colony vortex, with clear edges and tight arrangement (Figure 2A). To verify the multidifferentiation potential of human ADSCs, ADSCs were cultured in induction differentiation medium. The presence of red lipid droplets after Oil Red O staining confirmed their lipid differentiation ability (Figure 2B). The presence of red calcium nodules after Alizarin Red staining demonstrated osteogenic differentiation (Figure 2C) and Alcian Blue staining demonstrated chondrogenic differentiation (Figure 2D).

### Characterization of ADEs

**FIGURE 3:**
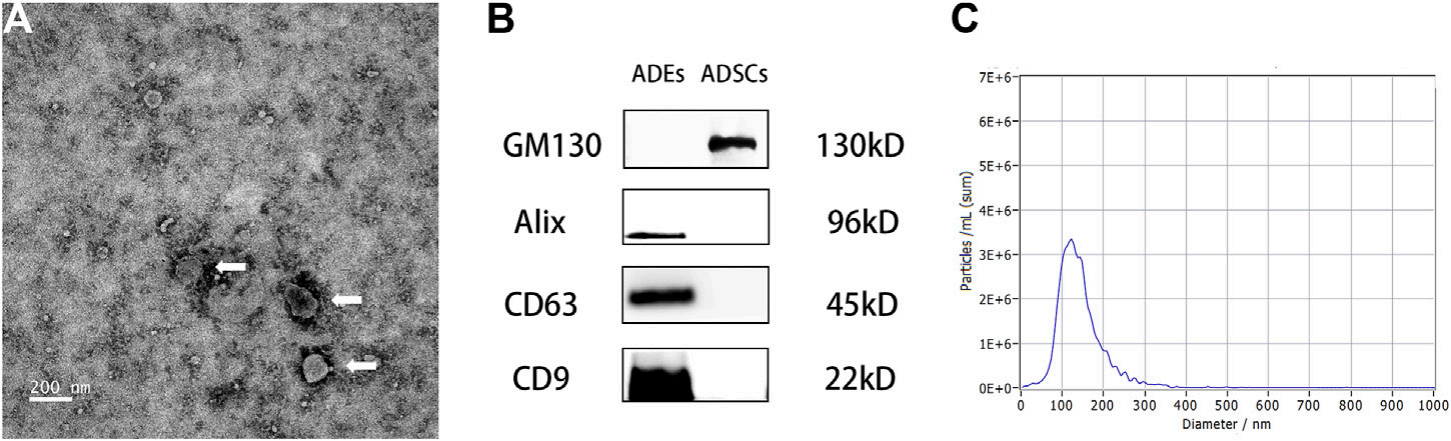
Morphological observation of adipose-derived exosomes (ADEs) and identification of surface markers. **(A)** The arrow points to human ADEs. **(B)** Identification of the surface-specific proteins CD63, CD9, and Alix in human ADEs. **(C)** Nanoparticle Tracking Analysis of ADEs.

The ADEs obtained from the ADSC medium were observed by TEM. The ADEs were round with clear edges and had a diameter of approximately 100 nm (Figure 3A). Western blot (Figure 3B) showed that the exosomal marker proteins CD63, CD9, and Alix were highly expressed, whereas the non-exosomal marker protein GM130 was not expressed in ADEs. NTA showed that the average diameter of ADEs was 130.5 ± 5.53 nm (Figure 3C). These results were all consistent with the characteristics of ADEs.

### Effect of ADEs on Relative mRNA Expression in the Hh Signaling Pathway

**FIGURE 4:**
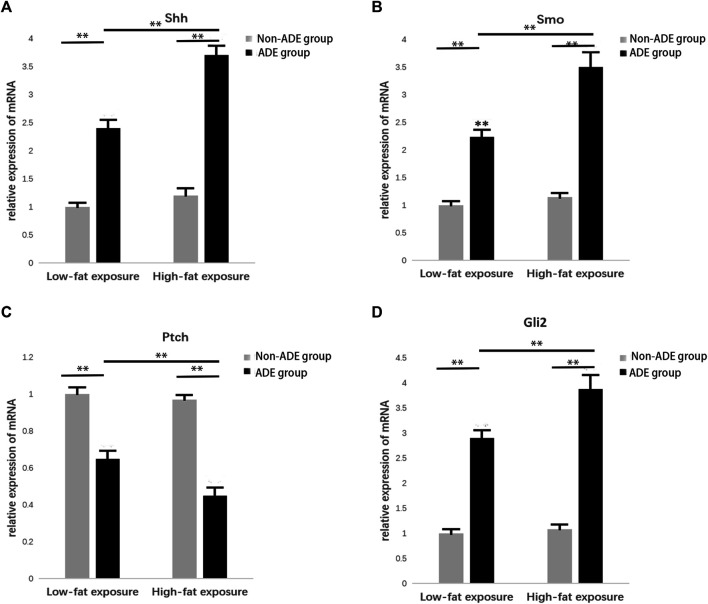
Gene expression in the Hedgehog signaling pathway 7 days after treatment with ADEs. **(A)** Shh expression was increased in the ADE group compared to the non-ADE group. **(B)** Smo expression was increased in the ADE group compared to the non-ADE group. **(C)** Ptc1 expression was decreased in the ADE group compared to the non-ADE group. **(D)** Gli2 expression was increased in the ADE group compared to the non-ADE group. *p < 0.05 and **p < 0.001. Shh, Sonic hedgehog; Smo, Smoothened; Ptc1, Patched 1; and Gli2, glioma-associated oncogene protein 2.

RNA was extracted from ADSCs treated with or without ADEs for 7 days to detect the relative expression of key genes in the Hh signaling pathway, including Sonic Hedgehog (*Shh*)*, Smo, Ptc,* and *Gli2*, using qRT-PCR. The relative expression levels of *Shh*, *Smo*, and *Gli2* were significantly increased in the ADE group in comparison with the non-ADE groups (*p* < 0.05; Figures 4A,B,D). Meanwhile, the expression level of the suppressor gene *Ptc1* was decreased in ADE group compared to the non-ADE group (*p* < 0.05; Figure 4C). Moreover, the expression of active genes and that of inhibitive genes in the high-fat exposure group significantly increased and decreased, respectively compared with that in the low-fat exposure group (Figure 4).

### Effect of ADEs on Adipogenic Differentiation of ADSCs

**FIGURE 5:**
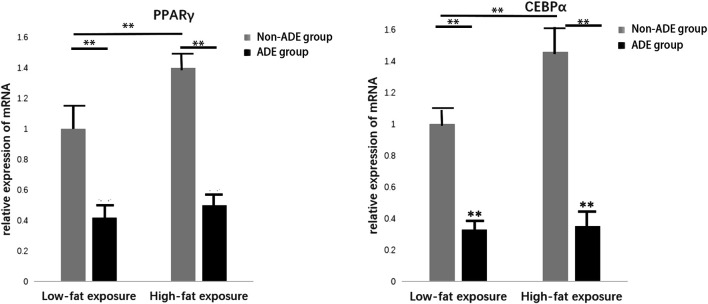
Expression of constituent lipid markers 14 days after adipogenic induction. Expression of PPARγ and C/EBPα was decreased in the ADE group compared to the non-ADE group and was increased in the low-fat exposure group compared to the high-fat exposure group when treated without ADEs. *p < 0.05 and **p < 0.001.

Induction of adipogenic differentiation was performed in each group to further verify the expression of related proteins during adipogenic differentiation of ADSCs. Cell proteins were extracted 21 days after induction, and the lipid differentiation markers peroxisome proliferator-activated receptor gamma (PPARγ) and CCAAT-enhancer binding protein alpha (C/EBPα) were detected. It was observed that protein expression in the high-fat exposure group was significantly higher than that in the low-fat exposure group, indicating that the exposure to high fat significantly promoted the adipogenic differentiation of ADSCs. Meanwhile, the lower protein expression in the ADE group than in the non-ADE group suggests that ADEs inhibit lipid differentiation (Figure 5).

### Effect of ADEs on Lipid Droplet Formation in ADSCs

**FIGURE 6:**
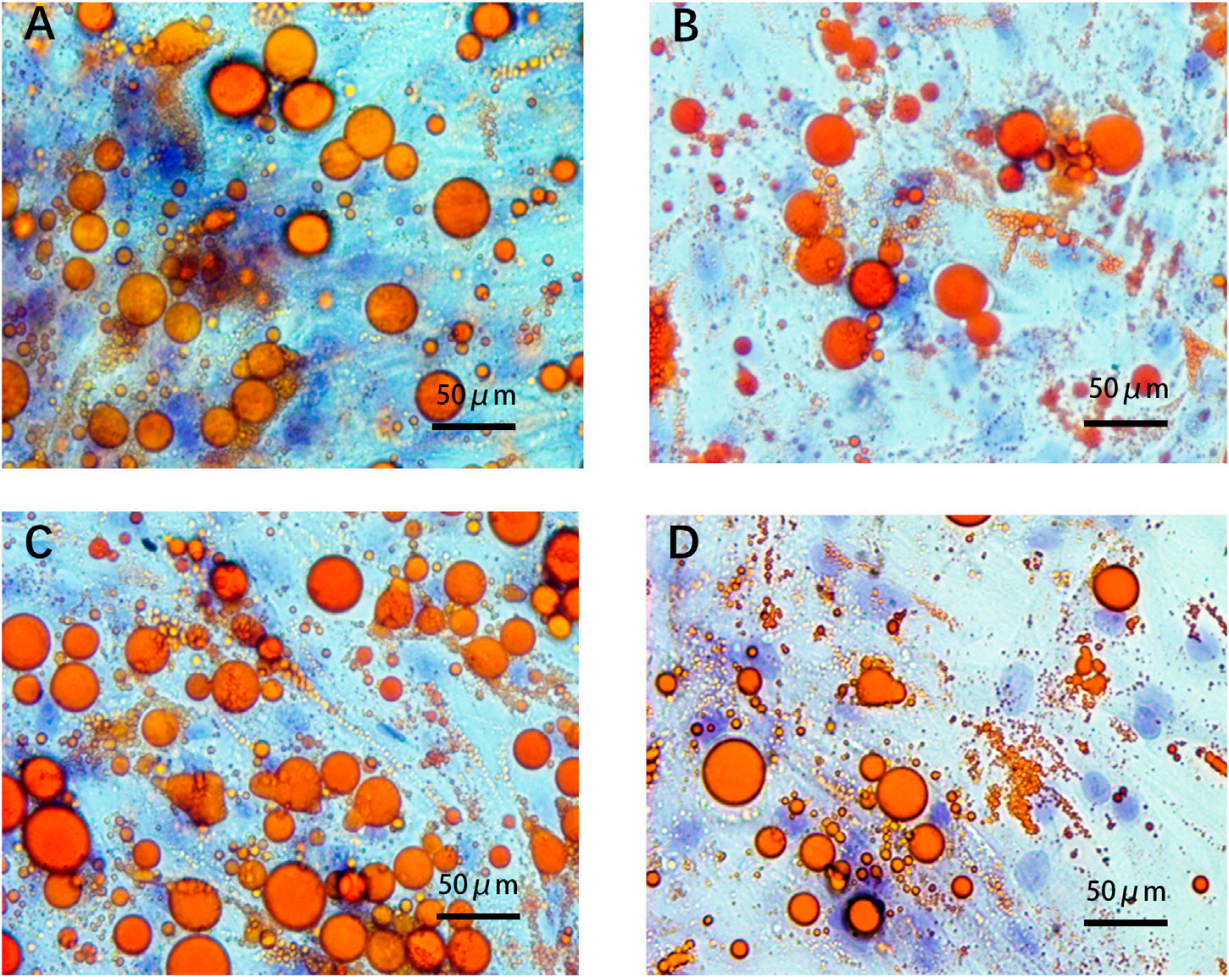
ADSCs assessed using Oil Red O staining. **(A)**. Low-fat non-ADE group; **(B)** Low-fat ADE group; **(C)** High-fat non-ADE group; and **(D)** High-fat ADE group. Groups treated with ADEs had fewer and smaller lipid droplets than the non-ADE groups.

**FIGURE 7:**
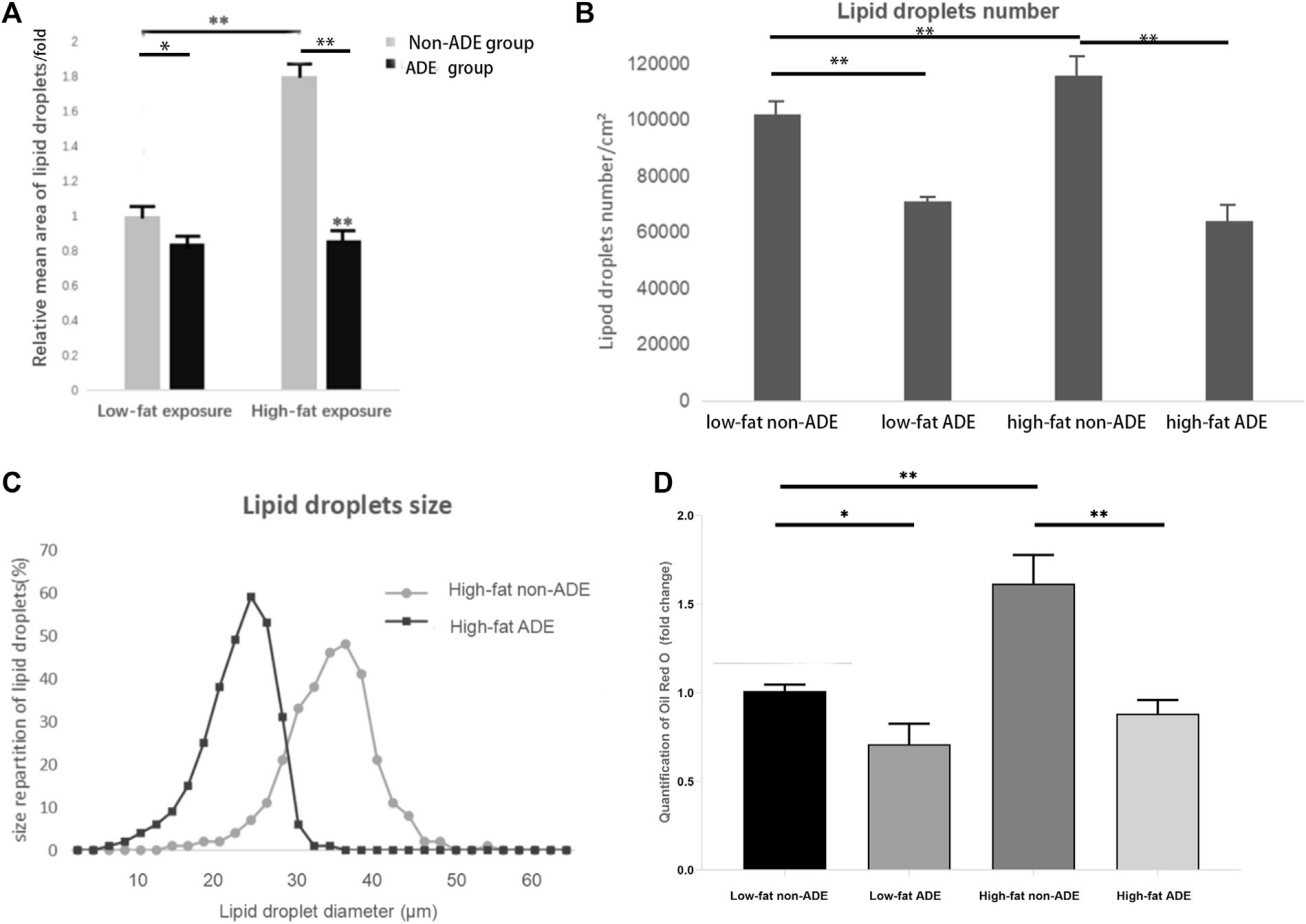
Comparison of lipid droplets between groups. **(A)** Mean size of at least 300 lipid droplets randomly chosen per condition were determined using ImageJ. **(B)** Mean number of lipid droplets per cm^2^. **(C)** Surface area of at least 300 lipid droplets. **(D)** Quantification of Oil Red O (fold change). *p < 0.05 and **p < 0.001.

After adipogenic differentiation, the adipocytes in each group were stained with Oil Red O (Figure 6). Three fields were randomly selected from each group per well, and ImageJ was used to statistically analyze the differences in lipid droplet formation between the groups. The presence of ADEs reduced the size and number of lipid droplets (Figures 7A,B,D). ADSCs co-cultured with ADEs showed a significant reduction in lipid droplet size and number when adipogenic differentiation was induced compared to the non-ADE group, especially in the high-fat exposure group. ADSCs under high-fat exposure tended to form larger and more lipid droplets compared to those under low-fat exposure. Quantitative analysis of lipid droplets showed that adipogenic differentiation in ADSCs was inhibited with the treatment of ADEs. The size distribution of lipid droplets in ADSCs with high-fat exposure are presented in Figure 7C.

## Discussion

In the present study, we revealed that ADEs could inhibit the adipogenic differentiation of ADSCs and that activation of Hh signaling is involved in this process. Upon ADE administration, the Hh signaling pathway was activated effectively and adipogenic differentiation was inhibited, especially during high-fat exposure. Ng et al. (2014) revealed that exosomes mediate the long-term effects of Hh signaling. A recent study showed that Hh signaling significantly inhibited the accumulation of adipose tissue in mice fed a high-fat diet (Shi and Long, 2017). Based on the results of the current and previous studies, we proposed that Hh signaling is necessary but not sufficient to inhibit adipogenic differentiation in ADSCs mediated by ADEs. Furthermore, quantitative analysis of Oil Red O staining suggested that ADEs inhibit not only adipocyte differentiation, but also lipogenesis in ADSCs.

Adipogenesis is a coordinated process involving many cell types and signaling molecules. The differentiation of ADSCs into adipocytes is closely related to their microenvironment (Rey et al., 2019). As the precursor cells of adipocytes, ADSCs play an indispensable role in lipid metabolism, especially in the microenvironment that affects lipid formation through paracrine signaling (Wu et al., 2015). Lipid synthesis mainly includes the *de novo* synthesis of saturated fatty acids, extension of the carbon chain of the fatty acids, and generation of unsaturated fatty acids. In the process of lipid synthesis, the enzyme glycerophosphate transacylase catalyzes the formation of phosphatidic acid using glycerol-3-phosphate and two molecules of lipoyl-CoA, which is then converted to diacylglycerol by phosphatase and finally generates fat *via* diacylglycerol transacylase (Blassberg and Jacob, 2017). Therefore, we added an appropriate amount of palmitic acid to the experimental groups to improve diacylglycerol generation and enhance lipid synthesis. Hh signaling exerts a crucial function by regulating lipid production in different organs during embryogenesis as well as in adults (Shi and Long, 2017). Shh signal transduction begins with lipid-modified Hh ligands, including SHH, Indian Hedgehog, and Desert Hedgehog. The receptor Ptc is composed of a single peptide chain in 12 transmembrane regions that can directly bind to the ligand and negatively regulate the Hh signal. The activation of Smo permits the subsequent activation of the transcription factor Gli2. Gli2 is a zinc finger protein that is the most downstream transcription factor of the Hh signaling pathway. Hence, the presence of *Gli* mRNA is a marker of Hh signaling pathway activation.

Exosomes are small vesicles capable of carrying the Hh protein (McGough and Vincent, 2016). They are highly valuable in the medical field, as they exert long-lasting effects, present no immunogenicity, and barely cause any trauma through noninvasive injection. Exosomes from adipose tissue have been confirmed to be associated with obesity and diabetes (Kranendonk et al., 2014). The present study demonstrates the association of exosomes from adipose tissue and adipogenesis *in vitro*, complementing the results of previous *in vivo* studies (Shi and Long, 2017). Previously, it has been reported that human ADSCs can absorb exosomes within a short period of time (Ashmwe et al., 2018). During this period, a large number of exosomes gather around the cellular nucleus, and some of them even enter the nucleus, which might account for the impact of ADEs on ADSCs.

Some studies have revealed that patients with obesity on a high-fat diet release more Hh protein owing to insulin resistance (Song et al., 2018). However, in the present study, the activation of the Hh signaling pathway by high-fat exposure was not significant. This may be because of the lack of a complete negative feedback loop in *in vitro* conditions, leading to the weak activation effect of high-fat exposure on Hh signaling. Even *in vivo*, activation of Hh signaling through insulin resistance did not counteract fat accumulation during high-fat diet. The high expression of *Shh*, *Smo*, and *Gli2* in the ADE groups suggests that Hh signaling is significantly activated under the synergistic action of ADEs. At the same time, the high-fat exposure enhanced the ADE-dependent activation of Hh signaling. The combination of high-fat exposure and ADEs effectively inhibited lipid accumulation during adipogenic differentiation of ADSCs.

The later stages of adipocyte differentiation are controlled by PPAR and C/EBP, two transcription factors that positively regulate each other. PPAR is a ligand-activated transcription factor that plays a key role in glycolipid metabolism, especially in the transcriptional regulation of fat-related genes in insulin-producing cells (Fontaine et al., 2008). C/EBP is another key regulator of adipogenic differentiation that is involved in several important cellular functions, such as cell proliferation and differentiation, tumorigenesis and apoptosis, and cell cycle regulation. In the present study, the expression of PPAR and C/EBP in ADSCs in the ADE groups decreased significantly during adipogenic differentiation, indicating that adipogenic differentiation of ADSCs was inhibited by ADEs. This result is consistent with the increased expression level of the Hh signaling pathway. The results of the present study suggest that Hh activation by a negative feedback loop triggered by a high-fat diet is not sufficient to counteract increased lipid accumulation.

Oil Red O staining showed that fat cells in the high-fat exposure groups were generally larger in size. Compared to the non-ADE group, lipid droplet formation in the ADE group was significantly inhibited by the activation of the Hh signaling pathway. The increase in fat detected was mainly because of the larger size of the fat cells, as bigger cells can harbor larger fat droplets. In adults with obesity, fat cell enlargement is the primary cause of weight gain. In the high-fat exposure groups, ADSCs accumulated significantly more lipids during adipogenic differentiation, and ADEs effectively reduced this effect. However, in the low-fat exposure groups, ADEs were not effective in reducing lipid droplet size and number. Our results suggest that the Hh signaling pathway can be effectively activated by the co-culture of ADEs and ADSCs. Adipogenic differentiation and lipid droplet accumulation in ADSCs exposed to high fat were effectively inhibited by ADEs. ADEs may exert the observed effect by preventing the accumulation of fat droplets rather than by directly reducing the size of the droplets.

Wound healing has always been the most challenging issue owing to the presence of various cells and molecules working in an orchestrated manner. Based on the fact that obesity impairs wound closure, in our study, we revealed that ADEs could inhibit adipogenic differentiation of ADSCs involving activation of Hh signaling. This selective inhibition has great potential for partial plastic surgery. However, whether this effect of ADEs is active *in vivo* remains to be determined. Further studies should be conducted to investigate the inhibition of ADEs during adipogenic differentiation *in vivo*. Exosomes from adipose tissue should be further explored as an important part of regenerative medicine for skin wound healing.

## Data Availability

The original contributions presented in the study are included in the article/supplementary material, further inquiries can be directed to the corresponding author.
